# Editorial: Application of Omics Technologies to improve robustness and resilience in livestock species

**DOI:** 10.3389/fvets.2023.1224630

**Published:** 2023-07-04

**Authors:** Marina Rufino Salinas Fortes, Sara Pegolo

**Affiliations:** ^1^School of Chemistry and Molecular Biosciences, The University of Queensland, Brisbane, QLD, Australia; ^2^Department of Agronomy, Food, Natural Resources, Animals and Environment, University of Padova, Padova, Italy

**Keywords:** livestock (including poultry), genomics, genetics, selective breeding, pathogens, disease resistance

Omics Technologies, such as genomics, transcriptomics, or metabolomics, are used to provide an understanding of the biological processes underlying phenotypes. They enable the annotation of genomes (1, 2), the identification of genetic variants (3), and the elucidation of complex molecular mechanisms (4, 5). Genomics has also transformed the practice of selective breeding livestock: the classic practice of selective breeding based on purely quantitative models and pedigree information changed to approaches that use genome-wide molecular information (6–8). This change in practice increased the accuracy of estimated breeding values. The application of Omics Technologies has the potential to improve the robustness and resilience of production animals to various environmental stressors and diseases.

Genomic selection, the practice of selecting animals based on their DNA profile (i.e., genotypes) (7), is successful in many traits and species. Genomic Selection can further develop to target “robustness” and “resilience” once these traits are defined as phenotypes linked to animal health and welfare (9). Resilience is the ability of an animal to adapt to various disturbances and return to its “normal” physiological state (10). Robustness can be defined as the ability to face environmental challenges while maintaining high productivity, without compromising reproduction, health, and welfare (11). In short, robustness implies that the animal maintains its functionality during a stressor event, which includes resilience. Omics Technologies can improve robustness and resilience by identifying genetic variants associated with disease or parasite resistance (12). These variants can be used in Genomic Selection, which can target multiple traits to improve overall performance. In addition, genomic data can inform mating systems to promote biodiversity, while selecting for enhanced performance. Genetic biodiversity should have a positive impact on animal resilience to pathogens.

In this Research Topic, there are five articles that showcase Omics Technologies applied to livestock research. The technologies featured include transcriptomics, metabolomics, and the development of a data-driven approach to improve animal husbandry. In their paper proposing the new data-driven approach to animal health and welfare, Thomann et al. explored their methods in all of the most common livestock species: cattle, sheep, goats, pigs, and poultry. Other articles in this Research Topic focused on sheep, goats, and chickens, exploring species-specific Omics data. Overall, the research presented in this Research Topic contributes to the annotation of animal genomes.

Three articles report on transcriptome data of small ruminants, which are relevant to the functional annotation of the genomes in less studied species and breeds. Hosseini et al. studied the transcriptome of adipose tissue in fat-tail sheep. Dixon et al. studied sheep's response to nematode infections, discovering genes linked to parasite burden. Zhao et al. studied adipose tissue gene expression in newborn goats because fat protects them against the cold. These are examples of transcriptomics being used to identify gene expression patterns associated with specific traits. Such information can be used to elucidate molecular mechanisms underpinning complex traits and ultimately lead to breeding more robust and resilient livestock.

Metabolomics can provide insight into the biochemical pathways involved in various physiological processes. For example, it can identify metabolic pathways that are important for growth and development. Li et al. used a metabolomics approach to study bone quality in chickens, a trait that affects productivity. Recently, Dehau et al. reviewed the applications of Omics to poultry (13).

To fulfill its full potential, Omics Technologies will be applied to animals, including humans and plants (6, 14, 15). Improved crops lead to feed and food security. As we use Omics to target animal welfare, hoping to breed livestock that is resilient and less susceptible to disease, it is possible to draw parallels with the use of Omics in human health (16). In this context, the article from Zhao et al. pointed out the possibility to translate the results among different species. Therefore, the integration of Omics approaches is promising for livestock production, crops, and personalized medicine. Integration of Omics data to enhance livestock production benefits from international collaborations and large consortium efforts, such as FAANG (1, 2). The accumulation of knowledge about genome function creates opportunities to address the issues related to livestock disease and pathogens.

## Author contributions

MF and SP edited this Research Topic together and co-authored the editorial text. All authors contributed to the article and approved the submitted version.
